# Comparison of Gene Coexpression Profiles and Construction of Conserved Gene Networks to Find Functional Modules

**DOI:** 10.1371/journal.pone.0132039

**Published:** 2015-07-06

**Authors:** Yasunobu Okamura, Takeshi Obayashi, Kengo Kinoshita

**Affiliations:** 1 Graduate School of Information Sciences, Tohoku University, Sendai, Miyagi, Japan; 2 Institute of Development, Aging and Cancer, Tohoku University, Sendai, Miyagi, Japan; 3 Tohoku Medical Megabank Organization, Sendai, Miyagi, Japan; Ghent University, BELGIUM

## Abstract

**Background:**

Computational approaches toward gene annotation are a formidable challenge, now that many genome sequences have been determined. Each gene has its own function, but complicated cellular functions are achieved by sets of genes. Therefore, sets of genes with strong functional relationships must be identified. For this purpose, the similarities of gene expression patterns and gene sequences have been separately utilized, although the combined information will provide a better solution.

**Result & Discussion:**

We propose a new method to find functional modules, by comparing gene coexpression profiles among species. A coexpression pattern is represented as a list of coexpressed genes with each guide gene. We compared two coexpression lists, one from a human guide gene and the other from a homologous mouse gene, and defined a measure to evaluate the similarity between the lists. Based on this coexpression similarity, we detected the highly conserved genes, and constructed human gene networks with conserved coexpression between human and mouse. Some of the tightly coupled genes (modules) showed clear functional enrichment, such as immune system and cell cycle, indicating that our method could identify functionally related genes without any prior knowledge. We also found a few functional modules without any annotations, which may be good candidates for novel functional modules. All of the comparisons are available at the http://v1.coxsimdb.info web database.

## Introduction

With the sequencing of the human genome completed [1–3], the next step is to annotate all of the functional elements in the genome, to reveal the genomic content. In spite of intensive analyses using EST [4], CAGE [5] and/or comparative genomics [6–8], about half of the genes remain uncharacterized. Thus, the focus has shifted to the functional annotation of the genes [9, 10].

Although each gene has its specific function, complicated cellular functions are usually achieved by combinations of individual functions, as in the ribosome, which synthesizes proteins by the coordinated functions of many ribosomal proteins and RNAs. Metabolic pathways are also good examples of genes that work together to achieve various biological functions. Therefore, to understand the functional role of each gene, it is essential to find groups of genes working with the same timing, by identifying genes with functional relationships. [11]

Various kinds of relationships can be considered to identify the functional modules. Protein-protein interactions (PPI), obtained by high throughput experiments such as yeast two-hybrid methods [12], provide some of the most comprehensive interaction data [13,14], but they only cover the proteins with direct interactions. In other words, genetic interactions (e.g. transcription factor and target gene) and metabolic pathways are not included. Another way to infer gene networks is based on the manual curation of the literature [15]. This approach provides high quality interaction data, but is quite time consuming and requires large amounts of human resources.

DNA microarrays generate profiles of comprehensive gene expression patterns and their clustering [16,17] to detect functionally related genes. Since one gene expression profile only provides a snapshot of a cell state, many expression profiles are required to detect related genes with reliable accuracy. Currently, over ten thousand gene expression data points are available for some microarray platforms, and they have been used to identify genes [18], genetic interactions [19] and gene modules [20,21].

To detect the regulatory relationships among genes, coexpression is a popular and promising approach [20,22]. Coexpression is calculated from large amounts of expression data obtained by microarray [23] or RNA-seq [24] experiments, to detect the genes with similar expression profiles. In this study, we have focused on the microarray data, because the number of available microarray samples is about 10 times larger than that of RNA-seq experiments. RNA-seq has some advantages, in terms of the gene expression profile quality. However, the number of samples is also an important factor to identify good functional relationships between genes, because larger coverage of various conditions is necessary to detect subtle functional connections. According to the progress of several international projects, such as ENCODE [25], the amount of available expression data is rapidly increasing, but is still currently limited as compared with that of DNA microarrays. Our approach will be applicable to RNA-seq data in the future, when larger amounts are available.

For the identification of gene functions, sequence conservation is also very useful. Since comparative analyses of genome sequences have worked very well to identify new potentially functional elements, as in the recent comparisons of 29 mammalian genomes [8], such analyses are becoming a standard practice when new genome sequences are solved [6,7,26].

Since both gene expression and sequence conservation are useful to understand gene functions, the introduction of conservation into analyses of gene expression profiles should be promising. Su *et al*. [27] compared the human and mouse transcriptomes, and found similar gene expression profiles in the corresponding organs. More recently, Brawand *et al*. [28] reported that the main differences in gene expression are due to the lineage, the chromosomes, and the tissues. These approaches were very useful to characterize the functional relationships among genes over species, but a serious problem still exists in the consideration of the conservation of gene expression patterns. It is easy to obtain samples from similar organs, but the similarity may not always indicate the correspondence of the organs. It is almost impossible to obtain samples corresponding to the same type of cells in the same state.

To overcome this difficulty, some studies have proposed methods to match samples over species. Le *et al*. [29] developed a method to match experiments over species, by introducing a new distance function between the samples, and Wise *et al*. [30] tried to match experiments based on their descriptions along with the expression data. These methods may work well to find similar gene expression states, but they naively assume that homologous genes have similar expression profiles. As we describe in this paper, this assumption is not always true.

We now propose a new method to compare gene expression patterns without sample matching, to focus on the relationships among the genes in each species and to compare the relationships among species. In this approach, we assume that the interactions between genes are conserved over species, if the interactions are fundamentally important for the biological roles of the genes. More precisely, we introduced a new method to measure the coexpression similarities. We created gene networks based on the conserved gene coexpression to find the functional modules by using a graph community detection algorithm, and found some well-enriched functional gene modules without any prior knowledge.

## Results & Discussion

### Patterns of coexpression conservation

**Fig 1:**
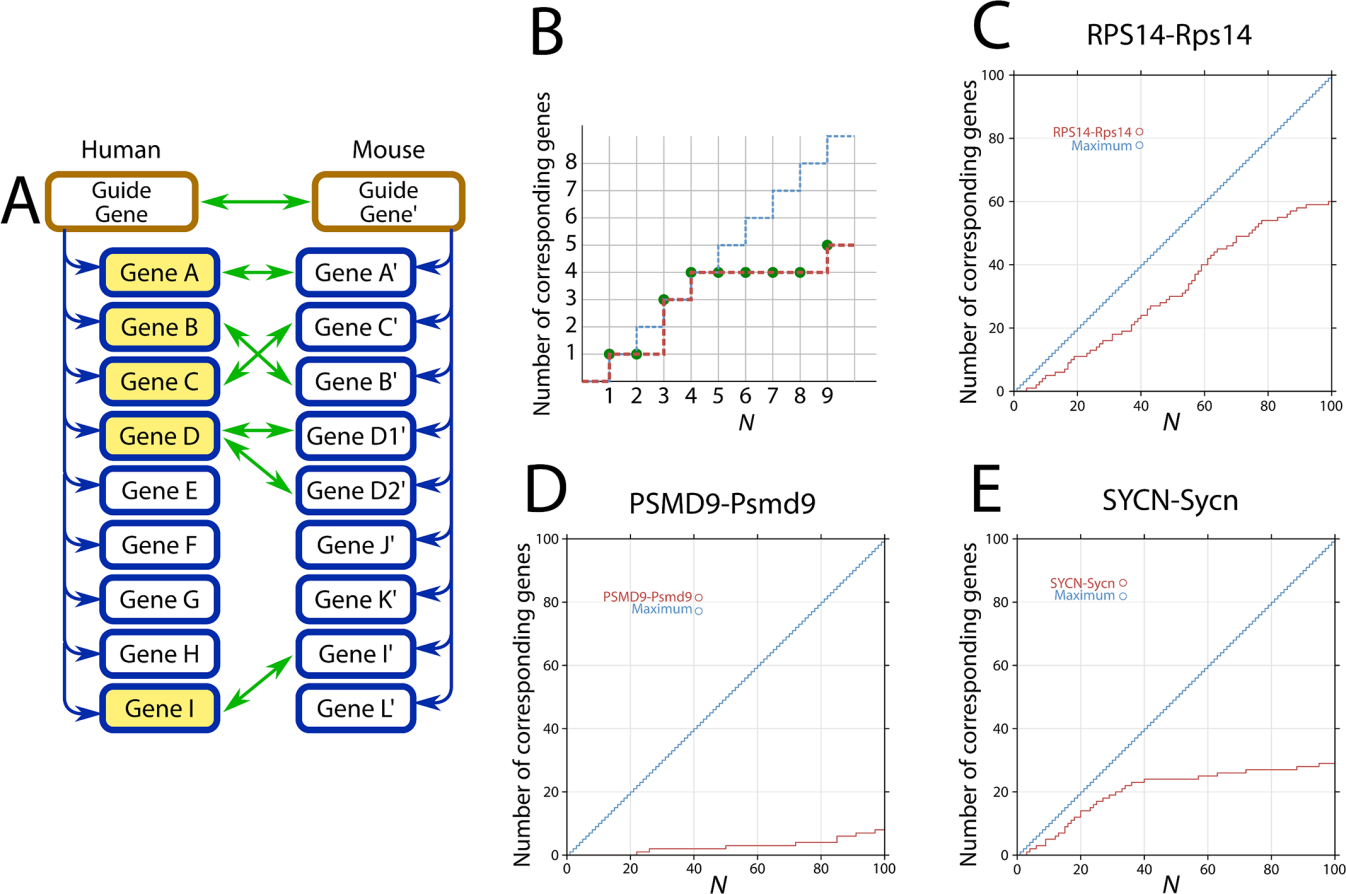
Overview of the conservation calculation method. (A) Schematic explanation of the comparison method for the conserved gene lists. Prepare a gene list pair for an orthologous gene pair from human and mouse. Count the number of human genes (yellow highlighted genes) with corresponding genes in the top *N* genes, where green arrows mean corresponding gene pairs. When a human gene corresponds to multiple mouse genes, we counted one human gene. However, when a mouse gene corresponds to multiple human genes, we counted all of the human genes. (B) Conservation chart of (A). This chart illustrates the change in the number of corresponding genes against the parameter value, *N*. (C) An example of a conservation chart for the most conserved guide gene. (D) An example of a conservation chart with a typical shape. (E) An example of genes with a turning point.

We compared the gene lists of the corresponding (or homologous) gene pairs to evaluate the conservation of coexpression patterns and expression data from two species, human and mouse. For each human gene (referred to as the guide gene), a list of coexpressed genes was created by ordering the genes by the coexpression strength, and a corresponding list of mouse genes was constructed for each homologous gene to the guide gene. The coexpression conservation of a homologous gene pair was measured as the similarity in the lists for the top *N* genes (Fig 1A). When the human guide gene had multiple homologous mouse genes, we compared the coexpressed gene lists for each pair of homologous genes. Next, we drew a “conservation chart” based on the number of corresponding gene pairs in the most coexpressed *N* genes, as shown in Fig 1B. If the human and mouse coexpression lists are exactly equal, then the conservation chart should look like the blue dashed line in Fig 1B. If the coexpression lists are equal to Fig 1A, then the conservation chart looks like the red dashed line in Fig 1B. A conservation chart represents the degree of similarity in the coexpression lists and indicates where the similarity exists.

One of the highly conserved genes was RPS14 (ribosomal protein S14), which had 71 corresponding genes in the top 100 most coexpressed genes (Fig 1C). Among the 60 genes, 55 are ribosomal genes, which correspond to 92% (= 55/60) of the human ribosomal genes tested. This result partially demonstrates the potential of our approach to detect related genes. However, many genes have low coexpression conservation, as in the example of PSMD9 (Fig 1D). On average, 13.1 genes were found to have corresponding genes in the top 100 most coexpressed genes.

Although the “shapes of the conserved lines” in the conservation charts were quite divergent and thus prevented a systematic classification, we found an interesting pattern, as shown in Fig 1E for SYCN (syncollin). This gene has a well-conserved region for the top 39 genes, while there were only slight increases after that, and 24 of the 39 genes have the homologous genes in mouse. SYCN is involved in the pancreatic secretion pathway (KEGG:hsa04972), and 12 of the 24 genes are also involved in the same pathway. This observation suggested that SYCN and the 24 genes may form a functional cluster for the pathway. When we assume that functional gene clusters are conserved over species, then the two coexpression lists for the orthologous gene should be similar over species. Therefore, it may be possible to detect the functional clusters by focusing on the well-conserved regions. Hereafter, we refer to the genes in conserved regions that have corresponding mouse genes (namely, the 24 genes in the above example) as “conserved coexpressed genes" or in short "CC genes".

### Identification of conserved coexpressed genes

To detect the CC genes from the conservation chart, we tried to identify a *turning point*, where a well-conserved region goes into a less conserved one. For this purpose, we searched for a point by detecting a flat region in each conservation chart, because a conservation chart should be flat for the genes in a list if the orders of the two coexpression lists are random. Thus, the initial point of the flat region was defined as the turning point, and we defined the conserved region as the part on the left of the flat area. The CC genes were identified as the corresponding genes between human and mouse of a guide gene in the conserved region. See the Materials and Methods section for the details of the turning point detection and the CC gene identification. As a result, 4,672 guide genes had a turning point. Each guide gene had 6.6 genes on average, and 3,776 non-redundant CC genes were identified.

### Conserved gene network in human

**Fig 2:**
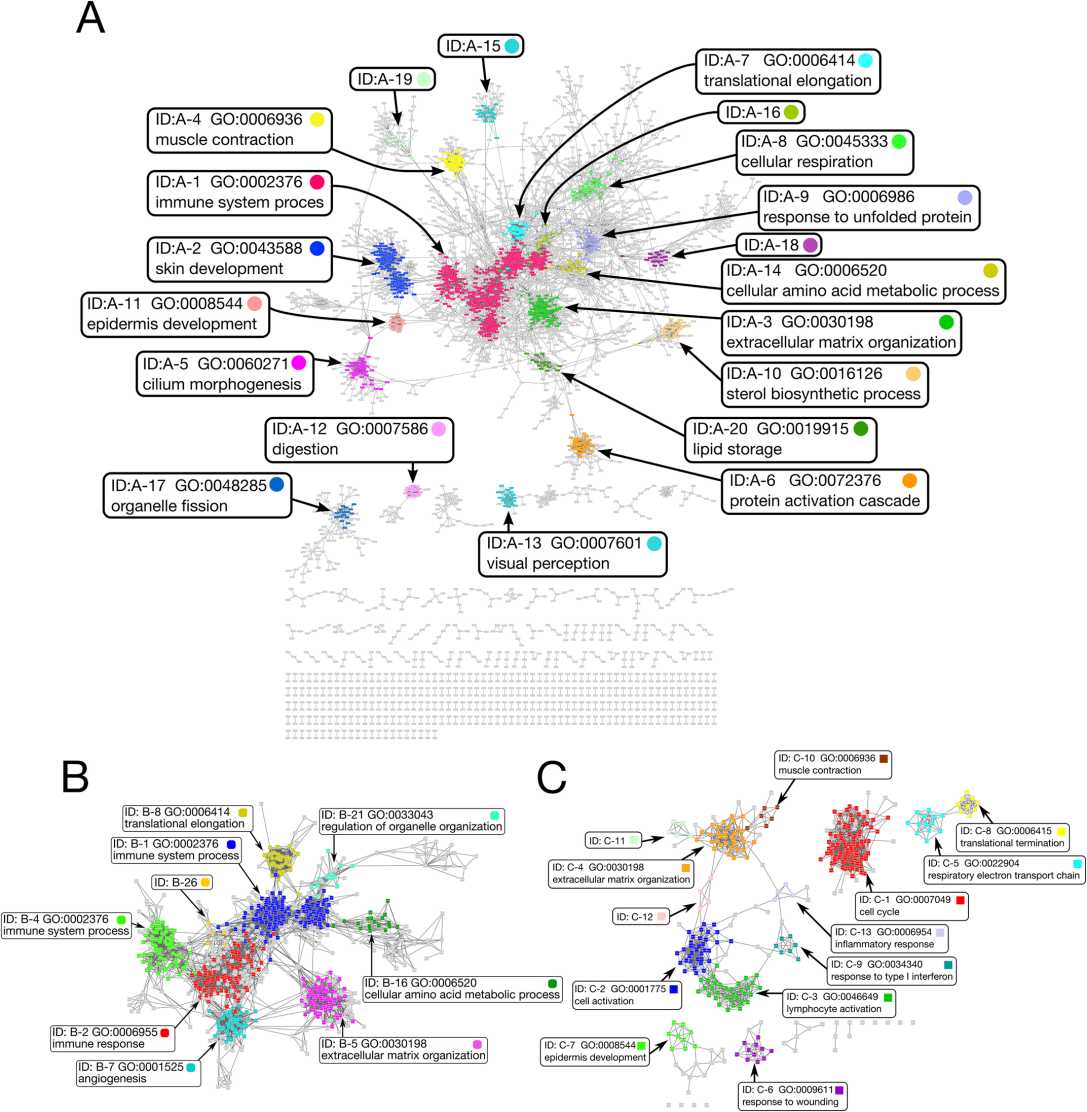
Detected gene networks (A) Gene networks based on coexpression conservation. We generated networks with 3,776 genes. The largest gene network contained 2,717 genes. Genes (nodes) were colored when they were a member of the top 20 largest modules with SCS = 4. Gray nodes were parts of some smaller modules, and black nodes were not parts of any modules. We prepared this picture of the network with Cytoscape ([31]). (B) The largest gene modules with SCS = 3 and the large modules with SCS = 5 are colored. This module has the representative term “immune system process”, but not all of the sub-modules with SCS = 5 have immune-related GO terms, as discussed in the text. (C) The gene network without a turning point. Since some gene networks had high coexpression conservation, no flat region was found. We used 100 instead of a turning point, because turning points cannot be defined for these genes. This network was generated from these highly conserved genes.

To visualize the relationships among all of the guide genes and their CC genes, we represented them in a network style, where each node corresponds to a gene and an edge is drawn from a guide gene to a CC gene, and removed all of the unidirectional edges. (Information about the nodes and edges is provided in the Cytoscape format in the S1 File.). The resulting networks are shown in Fig 2A. The networks consisted of one large and twenty small networks.

Since the large networks were too big to interpret, we separated them into more tightly related gene modules for convenience. For this purpose, we used the community detection algorithm developed by Palla *et al*. [32] for all of the networks shown in Fig 2A. This algorithm searches for densely connected sub-networks by integrating small cliques, and thus requires one parameter, the smallest clique size (SCS). We first used a default value (SCS = 4) and found 70 modules, as shown in Table 1. To characterize the functional roles of the modules, we performed GO enrichment analyses by the Fisher exact test, and selected the GO term with the smallest p-value from the statistically significant terms as the *representative GO* term. The genes in each module are shown in S1 Table.

**Table 1:** Detected gene modules. Summary of detected gene modules and representative GO terms when SCS = 4.

Community ID	Community Size	Representative GOID	Representative GO name	# of GO annotated	# of intersect	p-value
1	404	GO:0002376	immune system process	1897	232	1.14E-99
2	97	GO:0043588	skin development	295	27	2.32E-19
3	83	GO:0030198	extracellular matrix organization	353	32	2.81E-26
4	67	GO:0006936	muscle contraction	255	35	4.16E-40
5	48	GO:0060271	cilium morphogenesis	153	7	1.68E-02
6	43	GO:0072376	protein activation cascade	52	11	5.09E-14
7	42	GO:0006414	translational elongation	88	35	3.85E-70
8	32	GO:0045333	cellular respiration	145	25	6.40E-41
9	31	GO:0006986	response to unfolded protein	128	10	1.84E-09
10	28	GO:0016126	sterol biosynthetic process	48	18	1.55E-35
11	23	GO:0008544	epidermis development	256	8	8.33E-05
12	22	GO:0007586	digestion	107	8	4.98E-08
13	21	GO:0007601	visual perception	175	16	4.15E-23
14	19	GO:0006520	cellular amino acid metabolic process	430	15	7.19E-16
15	19					
16	18					
17	17	GO:0048285	organelle fission	496	12	2.77E-10
18	17					
19	16					
20	15	GO:0019915	lipid storage	57	6	3.59E-07
21	14	GO:0048706	embryonic skeletal system development	116	11	4.46E-17
22	13	GO:0006458	'de novo' protein folding	52	9	8.23E-16
23	12	GO:0034728	nucleosome organization	87	5	1.42E-04
24	10	GO:0030317	sperm motility	35	3	4.26E-02
25	10	GO:0045333	cellular respiration	145	8	9.16E-11
26	10	GO:0006936	muscle contraction	255	6	1.45E-04
27	9	GO:0007156	homophilic cell adhesion via plasma membrane adhesion molecules	91	9	2.48E-16
28	9	GO:0042438	melanin biosynthetic process	14	6	5.12E-13
29	8					
30	8					
31	7	GO:0006397	mRNA processing	393	7	2.62E-07
32	7	GO:0006096	glycolytic process	61	6	7.87E-10
33	6					
34	6	GO:0006956	complement activation	32	5	6.04E-09
35	6	GO:0031427	response to methotrexate	4	2	2.43E-02
36	6					
37	6					
38	6	GO:0043407	negative regulation of MAP kinase activity	65	4	1.50E-04
39	5	GO:0015988	energy coupled proton transmembrane transport, against electrochemical gradient	27	3	1.60E-03
40	5					
41	5	GO:0007588	excretion	63	3	2.16E-02
42	5	GO:0006364	rRNA processing	107	5	5.32E-07
43	5					
44	4	GO:0009954	proximal/distal pattern formation	29	4	3.52E-07
45	4					
46	4	GO:0002331	pre-B cell allelic exclusion	3	2	4.87E-03
47	4	GO:0006631	fatty acid metabolic process	296	4	4.65E-03
48	4					
49	4	GO:0008211	glucocorticoid metabolic process	24	4	1.57E-07
50	4					
51	4	GO:0006687	glycosphingolipid metabolic process	49	4	3.14E-06
52	4	GO:0007339	binding of sperm to zona pellucida	32	3	1.09E-03
53	4					
54	4					
55	4	GO:0006521	regulation of cellular amino acid metabolic process	60	4	7.23E-06
56	4					
57	4	GO:0022904	respiratory electron transport chain	93	3	2.83E-02
58	4					
59	4	GO:0006986	response to unfolded protein	128	4	1.58E-04
60	4					
61	4					
62	4					
63	4					
64	4	GO:0019322	pentose biosynthetic process	4	4	1.48E-11
65	4	GO:0060481	lobar bronchus epithelium development	5	2	1.62E-02
6	4	GO:0070059	intrinsic apoptotic signaling pathway in response to endoplasmic reticulum stress	29	3	8.00E-04
67	4					
68	4					
69	4					
70	4	GO:0002399	MHC class II protein complex assembly	4	2	9.74E-03

As a result, 45 of the 70 modules had significantly enriched GO terms. For example, the representative term of the largest modules shown as ID: A-1 in Fig 2A was GO:0002376 (immune system process), where 232 out of 404 genes had the GO term.

Some detected modules are not labeled with a Gene Ontology Term, as in the cases of the 15^th^, 16^th^, 18^th^ and 19^th^ modules. These modules had no significant terms with P-values < 0.05, and thus might be novel functional modules, such as the other modules with significant terms, because they have comparatively strong conserved coexpression.

Some gene modules had similar annotations and overlaps, indicating the existence of larger modules, if we searched modules for lower density. To elucidate the relationships among the modules, we observed the overlaps by changing three different SCS parameters of the module detection algorithm. We used three, four and five as the SCS to reveal both the low-density modules and high-density modules, as recommended by Palla *et al*. [32]. The numbers of detected gene modules were 107, 70 and 42, and the mean numbers of genes were 17.4, 19.3 and 24.6, respectively. The genes in the modules for SCS = 3 and SCS = 5 are shown in S2 and S3 Tables, respectively, and the enriched GO terms are shown in S4 and S5 Tables. The number of detected module with SCS = 4 (70 modules) may be larger than expected as expected, but it should be noted that our method will not detect the gene modules that were changed from mouse to mouse, because our method is based on the conservation between human and mouse, which may result in that the number of modules was limited.

The largest gene module in SCS = 3 is shown in Fig 2B. In this module, 308 out of 767 genes had the GO term GO:0002376 (immune system process). This module can be further separated into 9 sub-modules with 10 or more genes by using SCS = 5, as indicated in Fig 2B, where different colors represent the different modules with SCS = 5. Some of the colored gene modules were related to the immune system GO term, but others were not. For example, the ID: B-1, B-2 and B-4 gene modules in Fig 2B are related to GO:0002376 (immune system process), while the ID: B-5 gene module at the bottom right in Fig 2B with the representative GO: 0030198 (extracellular matrix organization), and some other enriched GO Terms as shown in the web database at http://v1.coxsimdb.info/coxsim/hsa-v13-01/mmu-v13-01/SCS:5/5. Most of the enriched GO terms are directly related with immune system process, but we can also see some interesting terms such as GO: 0032963 (collagen metabolic process) and GO: 0001568 (blood vessel development). This result may indicate that the immune system tightly cooperates with collagen metabolic process, blood vessel development and other systems.

Some genes lacked turning points and had large numbers of corresponding genes, indicating that the genes are quite strongly conserved. To characterize them, we generated another gene network for them by regarding 100 as the tentative turning point, instead of determining a turning point. As a result, 336 genes, 1,953 edges and 8 individual networks were detected (shown in Fig 2C). Only 9 genes among the 336 genes had no connection with other genes without any turning points. We applied the community detection algorithm again for this network, and found 13 modules. The largest module was ID: C-1 (Fig 2C), where 95 genes were involved and 85 of them were annotated as GO:0007049 (cell cycle). This result suggests that the genes for fundamental functions, such as cell cycle, translation or cytoskeleton, have highly conserved coexpression and are tightly connected in each function.

### Effect of the introduction of conservation

We performed the same module detection analysis for a human coexpression network without conservation, to evaluate the effect of the conservation. Coexpression data for human were obtained from COXPRESdb [33], where the strengths of coexpression are described by a rank-based measure called Mutual Rank (MR) [34]. Smaller MR values indicate stronger coexpression.

**Fig 3:**
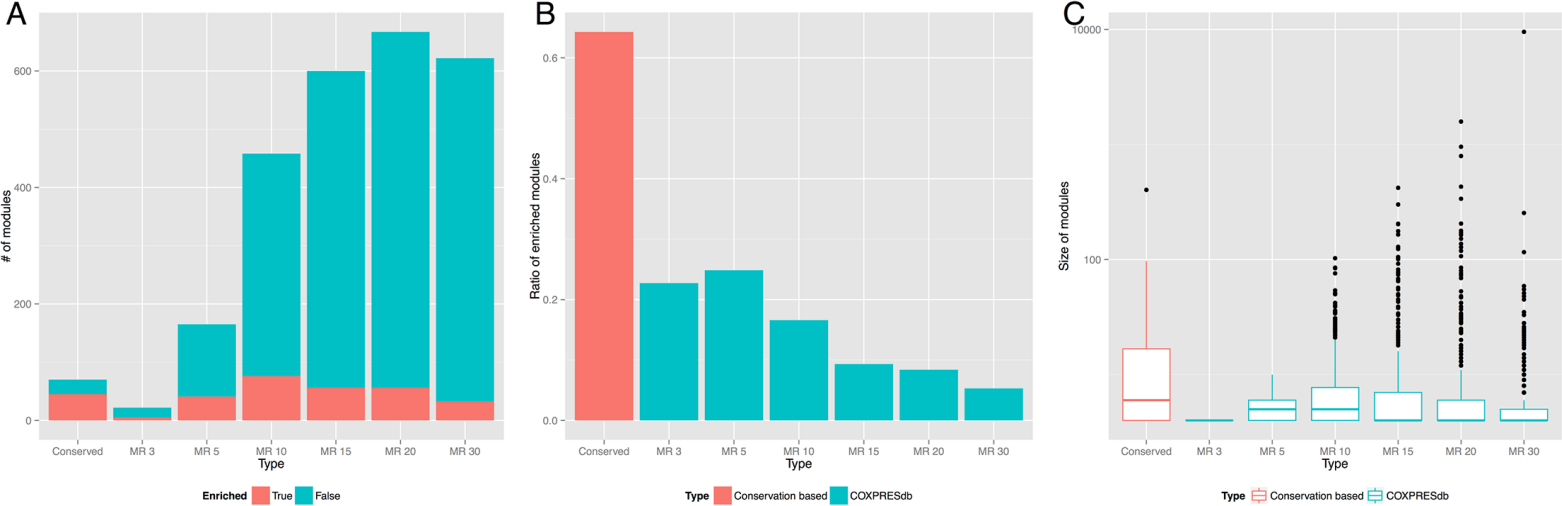
Comparison between the conserved coexpression-based modules and those based on coexpression without conservation. (A) The number of detected gene modules against MR for the coexpression-based method (left 6 bars) and the conservation-based method (right bar). The modules are colored according to whether a module had enriched GO terms. (B) The ratio of enriched gene modules. (C) A box plot of the gene module size distribution.

When we used MR = 3, 5, 10, 15, 20, and 30 as cutoffs, 22, 165, 458, 600, 667, and 622 modules were detected, respectively (shown in S6 Table). We calculated the GO enrichment of the modules for each MR threshold, and found that 5/22, 41/165, 76/458, 56/600, 56/667, and 33/622 modules were enriched with at least one GO term. However, the conservation filtering method proposed in this paper detected 45 enriched modules out of 70 modules (Fig 3A), and the ratio of enriched modules based on coexpression conservation is clearly better than the ratios of enriched modules based on the non-filtering method with COXPRESdb at any MR threshold (< 41/165 with MR = 5, see Fig 3B). This observation suggests that the conservation-based method may reduce false positives to identify functional modules.

To check the reduction of false positives in each module, we further compared the modules with MR = 10 (458 modules) and the modules identified by conserved coexpression (70 modules). We found that 47 modules were *similar* (S7 Table), where a pair of modules was judged to be similar if the number of common genes was significantly large (Fisher’s exact test, p-value < 0.05 with Bonferroni correction). If a module had multiple similar modules, then only the mutually best pair was used. We also counted the number of genes with the representative GO term of the module ($N_{rep\;GO}^{gene}$), and used the ratio to the number of genes in the module ($N_{rep\;GO}^{gene}/N^{gene}$) as an indicator to evaluate the goodness of the modules. If we assume that the representative GO term truly explains the function of a module, then a higher ratio indicates a better module explanation, or a module with fewer falsely related genes (or genes with different annotations). As a result, 13 out of 47 modules were found to share the same representative GO term (S7 Table), and the average ratio ($N_{rep\;GO}^{gene}/N^{gene}$) was 1.18 times higher in the conservation-based method than the COXPRESdb method. Notably, the raw number $N_{rep\;GO}^{gene}$ was also 1.18 times higher and the sizes of the conservation coexpression-based modules were larger than those of the COXPRESdb-based modules (Fig 3C), indicating that fewer falsely related genes were included in the modules (S7 Table).

**Fig 4:**
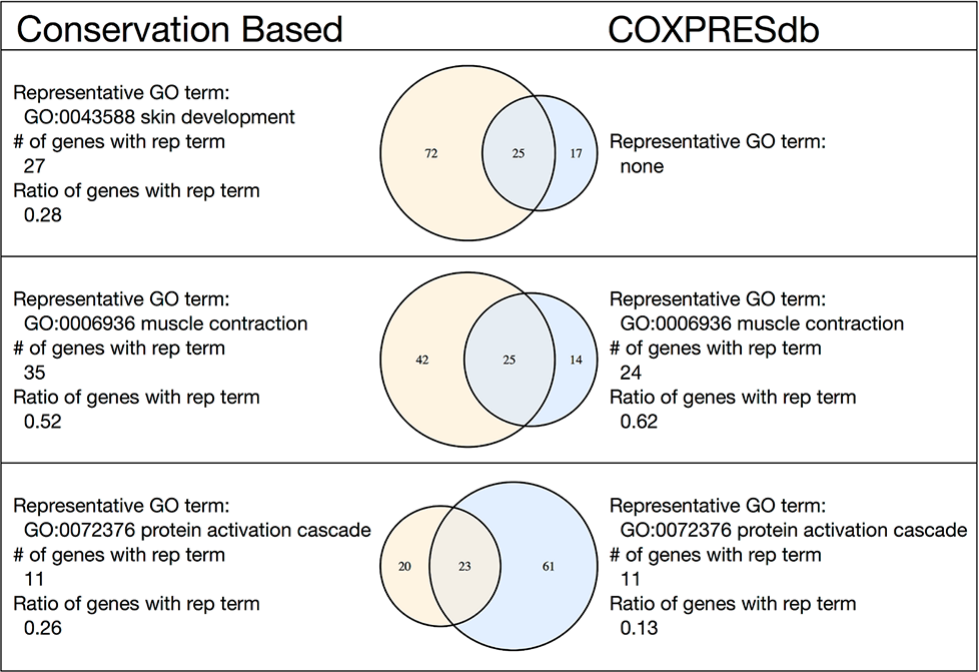
Example of the correspondence between the conservation-based method modules and the COXPRESdb-based modules. The three module pairs with the largest numbers of intersecting genes are shown. The list of all similar module pairs is provided in S7 Table.

Some examples of similar module pairs are shown in Fig 4. The first module pair in Fig 4 and S7 Table has different representative GO terms with 25 common genes, one for “skin development” and the other has no significant term, where the size of the conservation-based module (97 = 72+25) is much larger than that of COXPRESdb (42 = 25 + 17). The larger size and the existence of the representative GO term indicate the enhanced enrichment of the related genes. The second module pair also has a larger number of genes with the representative term in the conservation-based module (35) than that of COXPRESdb (24). Since it shares the same representative GO terms, the larger number of genes with the representative GO term may indicate the presence of a smaller number of related genes outside of the module. However, the ratio of the genes with a representative GO term for the conservation-based module (0.52) is smaller than that of COXPRESdb (0.62), which indicates the inclusion of a larger number of unrelated genes in the conservation-based modules. Since the conservation charts of the large module member genes have few flat regions in a small *N* range, the turning points of these genes were found in a large *N* range. Therefore, genes that are not directly related to a representative term may be included in the detected gene module. As described above, the conservation-based modules have better $N_{rep\;GO}^{gene}/N^{gene}$ ratios on average, as in the case of the third example. However, in some cases the COXPRESdb-based modules produce better modules from the viewpoint of the inclusion of falsely related genes, as in the second example. In short, coexpression conservation may reduce the number of false negatives and false positives, to detect the functionally related genes on average.

### Implementation of web-based database

**Fig 5:**
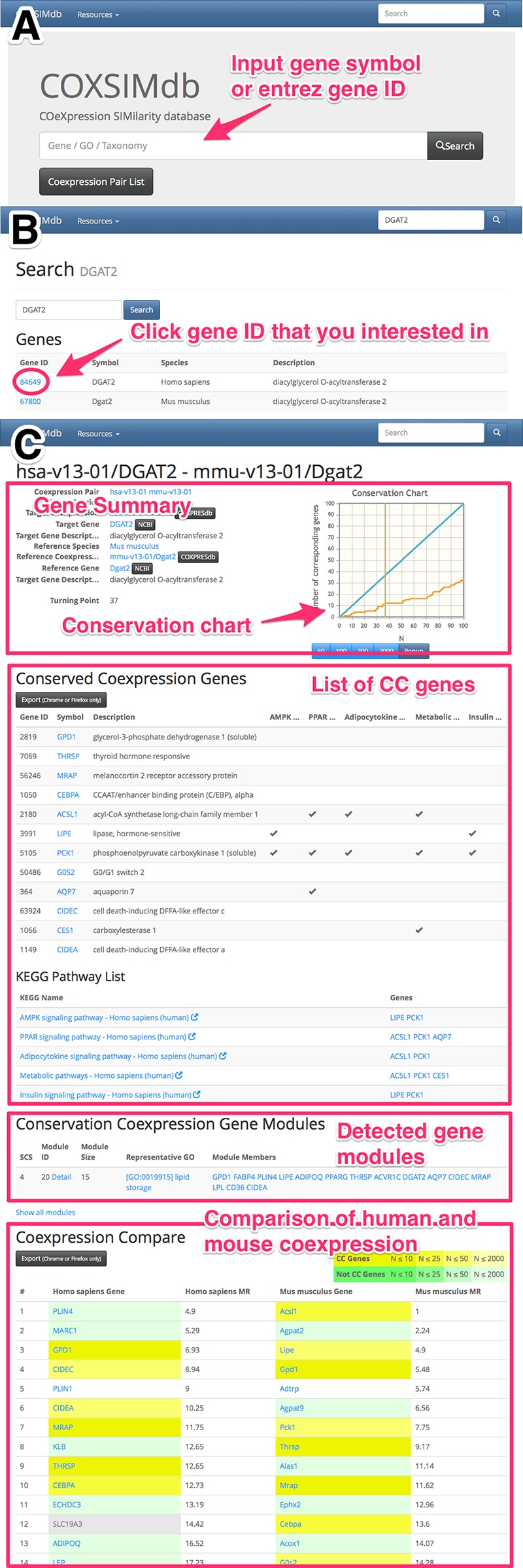
How to use COXSIMdb. **(A)** First, search for a gene by its symbol or entrez gene ID. **(B)** Second, select a gene of interest. **(C)** View of the coexpression conservation results. This view provides a summary of the genes, a list of CC genes, the detected gene modules, and a comparison of coexpression.

All results of coexpression conservation, CC genes, and module detection are available through the web database named **CO**e**X**pression **SIM**ilarity **D**ata**B**ase (COXSIMdb, http://v1.coxsimdb.info). The overview of the database is shown in Fig 5. To use this web database, insert the gene symbol or entrez gene ID into the search field at the top of the COXSIMdb page (shown in Fig 5A). This web service provides a list of genes related to the query, with a view of the results of the coexpression conservation of a gene (Fig 5B). Fig 5C illustrates an example of a COXSIMdb main result view. The result view has up to 4 sections. The first section is a summary of the human and mouse genes and a conservation chart. The second section is a list of CC genes and any associated KEGG pathway. The third section is a list of detected gene modules that include the gene if it is involved in the modules. The gene modules detected with SCS = 4 are shown in the default mode, but links to the modules detected with SCS = 3 and SCS = 5 are also provided. The last section is a table view of the comparison of coexpressed genes between human and mouse. Each gene is colored by the gene type and whether it is a CC gene, and homologous genes are shown in a pop-up window when the cursor moves over the genes.

## Conclusion

In this paper, we have described a new method to compare gene expression patterns by focusing on gene coexpression, to avoid the problem of sample matching. We also developed an algorithm to detect the conserved modules, and the GO term enrichment analyses revealed that the conserved gene modules have strong functional relationships. In other words, our method could detect some functional modules, without any prior knowledge. Many modules are well known, such as ribosomal protein or immune system, but some detected modules have significantly enriched GO terms, and thus they will be good candidates for further experimental analyses to identify the novel functional modules.

## Materials and Methods

### Datasets

All human and mouse coexpression data were obtained from COXPRESdb [33], versions Hsa.c4-1 (20,280 genes) and Mmu.c3-1 (20,959 genes), respectively. COXPRESdb is a database of co-regulated gene relationships. The coexpression strengths were obtained from COXPRESdb, and are represented by Mutual Rank (MR) [34]. MR is a rank-based measure, and smaller values indicate stronger coexpression. We prefer MR over the Pearson Correlation Coefficient (PCC), because MR shows better performance in GO prediction [34]. All homologous gene sets were obtained from HomoloGene [35], version build 65, and the genes that were not in HomoloGene were removed from the analyses. There were 18,981 human genes and 21,766 mouse genes in HomoloGene, and we used 14,611 homologous gene pairs between human and mouse in our analyses. We used Gene Ontology Terms (GO Terms) [36] to annotate the functions of the gene modules. The correspondence between the genes and the GO terms was obtained from the gene2go file in NCBI [35].

### Detection of turning point and conserved coexpression genes

As described in the Results and Discussion section, we counted the number of human genes with mouse homologs to draw the conservation chart (Fig 1A and 1B), and then searched for the lines with a turning point. It should be noted that we counted the number of human genes when a gene had multiple homologous genes in mouse. In other words, a human gene with two or more homologous genes in mouse was counted as one, while a mouse gene with two human homologs was counted twice.

In the example shown in Fig 1E, some conservation charts have two distinct regions, highly conserved and non-conserved, which can be detected as a turning point in the conservation chart. When a functional gene relationship is conserved between two species, the gene coexpression relationship will also be conserved. Therefore, to detect the functional modules, we tried to detect the turning point in each conservation chart.

The turning point is detected by focusing on the flat area in a conservation chart. If a gene module has *k* genes, then the two coexpression lists should have the same order in the top *k* genes, but the orders in the list after the *k* genes can be expected to be random. Therefore, if no new corresponding genes are found after the highly conserved region, it should be the turning point. We defined the turning point as the region with a 10-length flat region, which is a region with no new corresponding genes, and defined the conserved region as the region to the left of the turning point. We searched for turning points among the top 400 coexpressed genes.

When we also checked 5, 10, 15 and 20 as the length of the flat region to define the turning point, 1,890, 3,776, 3,478 and 2,783 non-redundant conserved-coexpressed genes (CC genes, as described below) were found, respectively. We selected the length of the flat region to maximize the number of CC genes. On the one hand, the use of flat regions longer than 10 to detect the turning point decreased the number of CC genes, because no flat region was found in the conservation chart. On the other hand, the shorter flat region also made the number of CC genes decrease, because turning points were found in the first position.

The genes in the conserved regions can be considered to have strong functional relationships. Therefore, we focused on the genes in the conserved regions, to emphasize their strong relationship with the guide gene. Since some unrelated genes can be mixed in the coexpression lists due to coexpression noise, we used the genes *mutually* found in the conserved regions and named them CC genes. In other words, if gene A is the CC gene of guide gene B, then guide gene B should also be a CC gene of gene A. If there were multiple turning points, our turning point detection algorithm selected the first one of them, and tended to select the turning point at the smallest *N*.

Some genes did not have a flat area because their coexpression lists were highly conserved. We also generated a conserved coexpression gene network by using the following method. Since these genes did not have a flat area, we could not determine a turning point. We used 100 as the threshold of *N* instead of the turning point in these cases. Subsequently, we generated a coexpression gene network without a flat area, using the same procedure described above.

A Python implementation to calculate the conservation and the turning point is available at S2 File.

### Analysis of the gene network and module detection

Since the CC genes are those with a tight functional relationship to the guide gene, we represented the relationship as a network, where a node indicated a gene and an edge represented a relationship between a CC gene and the guide gene.

Biological networks tend to be scale-free, with a small world network and a modular structure [37–39]. Since our network also had similar features, we applied a community detection algorithm implemented in networkx [40] to find the functional modules, according to Palla *et al*. [32]. To characterize the functional roles of the modules, enrichment analyses were performed, using TargetMine [41] and based on Fisher’s exact test. We defined the *representative GO* term as the GO term with the smallest p-value in a module.

Since some gene modules had overlaps or similar annotations, we performed the module detection with three different strictness values, corresponding to the change in a parameter for the smallest clique size (SCS) used in Palla *et al*. [32]. Detection with a larger SCS yielded smaller and higher clustering coefficient modules. More precisely, we used three, four and five for the three different SCS values, and calculated the overlaps between the detected gene modules. Finally, we performed clustering of the gene modules by connecting the overlapped modules.

## Supporting Information

S1 File The edge list of the network shown in Fig 3. Since this network is an undirected graph, we did not distinguish column 1 from column 2.
(ZIP)

S2 File A Python implementation to calculate the conservation and the turning point. (GZ)

S1 Table The list of genes in detected clusters when SCS = 4. (XLSX)

S2 Table The list of genes in detected clusters when SCS = 3. (XLSX)

S3 Table The list of genes in detected clusters when SCS = 5. (XLSX)

S4 Table The list of representative GO terms when SCS = 3. (XLSX)

S5 Table The list of representative GO terms when SCS = 5. (XLSX)

S6 Table The list of genes in detected modules in COXPRESdb-based analysis when SCS = 4. (CSV)

S7 Table The correspondence between conservation coexpression-based modules and mutual rank-based modules. (XLSX)
